# Functional characterization of two members of histidine phosphatase superfamily in *Mycobacterium tuberculosis*

**DOI:** 10.1186/1471-2180-13-292

**Published:** 2013-12-11

**Authors:** Olabisi Oluwabukola Coker, Saradee Warit, Kamolchanok Rukseree, Pijug Summpunn, Therdsak Prammananan, Prasit Palittapongarnpim

**Affiliations:** 1 Department of Microbiology, Faculty of Science, Mahidol University, Rama 6 Road, Bangkok 10400, Thailand; 2 National Center for Genetic Engineering and Biotechnology, National Science and Technology Development Agency, PathumThani 12120, Thailand; 3 Current Address: Mahidol University, Amnatcharoen Campus, Muang, Amnatcharoen 37000, Thailand; 4 Current Address: School of Agricultural Technology, Walailak University, Nakhon Si Thammarat 80161, Thailand

**Keywords:** Rv2135c, Rv0489, Acid phosphatase, Hypothetical protein, Histidine phosphatase superfamily, Phosphoglycerate mutase, *Mycobacterium tuberculosis*

## Abstract

**Background:**

Functional characterization of genes in important pathogenic bacteria such as *Mycobacterium tuberculosis* is imperative. Rv2135c, which was originally annotated as conserved hypothetical, has been found to be associated with membrane protein fractions of H37Rv strain. The gene appears to contain histidine phosphatase motif common to both cofactor-dependent phosphoglycerate mutases and acid phosphatases in the histidine phosphatase superfamily. The functions of many of the members of this superfamily are annotated based only on similarity to known proteins using automatic annotation systems, which can be erroneous. In addition, the motif at the N-terminal of Rv2135c is ‘RHA’ unlike ‘RHG’ found in most members of histidine phosphatase superfamily. These necessitate the need for its experimental characterization. The crystal structure of Rv0489, another member of the histidine phosphatase superfamily in *M. tuberculosis*, has been previously reported. However, its biochemical characteristics remain unknown. In this study, Rv2135c and Rv0489 from *M. tuberculosis* were cloned and expressed in *Escherichia coli* with 6 histidine residues tagged at the C terminal.

**Results:**

Characterization of the purified recombinant proteins revealed that Rv0489 possesses phosphoglycerate mutase activity while Rv2135c does not. However Rv2135c has an acid phosphatase activity with optimal pH of 5.8. Kinetic parameters of Rv2135c and Rv0489 are studied, confirming that Rv0489 is a cofactor dependent phosphoglycerate mutase of *M. tuberculosis*. Additional characterization showed that Rv2135c exists as a tetramer while Rv0489 as a dimer in solution.

**Conclusion:**

Most of the proteins orthologous to Rv2135c in other bacteria are annotated as phosphoglycerate mutases or hypothetical proteins. It is possible that they are actually phosphatases. Experimental characterization of a sufficiently large number of bacterial histidine phosphatases will increase the accuracy of the automatic annotation systems towards a better understanding of this important group of enzymes.

## Background

*Mycobacterium tuberculosis* remains a threat to global health despite efforts directed towards its eradication. Although several works have been done in recent years towards understanding the genetic repertoire of this organism, many of its strategies involved in virulence, pathogenesis and resistance to both host pressure and antibiotics remain elusive [1]. Mycobacterial genome has been completely sequenced for over a decade [2]. However, the functions of many of its genes are annotated based only on similarity to known proteins using automatic annotation systems. This method of function annotation can be erroneous [3,4]. Errors in automatic function annotation to genes in bacterial genomes are well documented. They often lead to misinformation that may hamper the understanding of the roles played by many bacterial genes [5-8]. Experimental characterization of additional mycobacterial proteins is needed to aid deeper understanding of the organism.

Histidine phosphatase superfamily is a large family of proteins with diverse functions that are important. This superfamily comprises two branches. The larger branch consists of proteins which function in metabolic regulations, intermediary metabolism and developmental processes. Examples include cofactor dependent phosphoglycerate mutases, alpha-ribazole phosphatase (CobC), mannitol-1-phosphatase, fructose-2,6-bisphosphatase and acid phosphatase (PhoE) [9-11]. The smaller branch consists mainly of phosphatases and phytases with functions ranging from extracellular metabolism to involvement in developmental processes [9,12]. Examples include human testicular acid phosphatase and lysosomal acid phosphatase [9,13,14]. The functions of enzymes in this superfamily are based on a conserved catalytic histidine residue in the motif ‘RHG’ present at the N terminal, which becomes phosphorylated during the reaction [9,15].

Members of the histidine phosphatase superfamily that have been studied in *M. tuberculosis*, include Rv0489. The crystal structure of Rv0489 at 1.7 Å resolution reveals the catalytic residues superimposing with those of the cofactor dependent phosphoglycerate mutase of *E. coli*, with which it shares 42% amino acid identity [16]. However, its biochemical characteristics remain unknown. Other members include Rv3214c, an acid phosphatase with unknown specific substrate [3] and Rv2419c which was characterized as glucosyl-3-phosphoglycerate phosphatase in lipopolysaccharide biosynthesis with an optimum pH of 7.0 [17]. Rv2135c is a paralog of the aforementioned members of the superfamily, but it is annotated as a hypothetical protein in the genomic database of *M. tuberculosis*[18]. Bioinformatics similarity searches show that it is a probable cofactor dependent phosphoglycerate mutase. However, there have been reports that proteins annotated as cofactor dependent phosphoglycerate mutases by sequence similarity actually perform the functions of an acid phosphatase when assayed *in vitro*[9]. Examples in *M. tuberculosis* are Rv2419c [17] and Rv3214c [3]. In other organisms, examples include PhoE of *Bacillus stearothermophillus*, and PfPGM2 of *Plasmodium falciparum*[4,19].

Rv2135c was found in Triton X-114 fractions of *M. tuberculosis* H37Rv strain and reported as one of the cell envelope associated hypothetical proteins [20]. Rv2135c contains a catalytic histidine motif similar to proteins in histidine phosphatase superfamily. Nevertheless, its motif is ‘RHA’ unlike ‘RHG’ commonly found in histidine phosphatase superfamily. These motivate the need to investigate its function in the metabolism of *M. tuberculosis*.

Phosphoglycerate mutases (EC 5.4.2.1) primarily interconvert 3-phosphoglyceric acid (3-PGA) and 2-phosphoglyceric acid (2-PGA) in both glycolysis and gluconeogenesis [12,21]. Two different types of phosphoglycerate mutase have been identified. One depends on the cofactor, 2,3-bisphosphoglyceric acid, for activity (dPGMs) while the other does not (iPGMs) [12,21]. The cofactor-dependent form is found in vertebrates, budding yeast, and bacterial species, while the cofactor-independent form is the only phosphoglycerate mutase present in higher plants. Some bacteria like *E. coli,* however, possess both forms [22]. There is no amino acid sequence similarity between these two types of PGMs and their structures are also quite different. Deficiencies in dPGM in *E. coli* and yeast have been linked to severely impaired growth [23,24].

Acid phosphatases (EC 3.1.3.2) catalyze the hydrolysis of phosphate monoesters or transfer of phosphate groups between phosphoester and alcohols. The enzymes catalyze optimally at acidic conditions and are completely and structurally different from alkaline phosphatases (EC 3.1.3.1), which work optimally at alkaline conditions [25-27]. Unlike the alkaline phosphatases, the acid phosphatases, do not utilize metal ions in their catalysis. They rather utilize histidine residue to form a phospho-histidine-enzyme intermediate which is essential for their catalysis. In contrast, alkaline phosphatases make use of a phospho-serine-enzyme intermediate for their catalysis and have a binuclear Zn (II) active site [26,28]. Phosphatases are important in the physiology of an organism as they function in many catalytic reactions relating to activation or deactivation of enzymes. Deficiencies in phosphate metabolism have been reported to be related to reduction of virulence in many bacterial species such as *Listeria monocytogenes*, *Streptococcus pneumoniae*, *Vibrio cholerae, Proteus mirabilis* and *M. tuberculosis*[29-34].

The fact that histidine acid phosphatases and cofactor dependent phosphoglycerate mutases share similar catalytic amino acid residues and mechanism of catalysis warrants their placement in the same superfamily [9]. This often leads to some difficulties in predicting the function of an enzyme that belongs to the superfamily. Thus, biochemical characterization of purified enzymes is necessary before the function of any member of histidine phosphatase superfamily can be ascertained. In this study, we report the first cloning, purification and characterization of *M. tuberculosis* Rv2135c. In addition, we cloned and characterized Rv0489. Its role as a cofactor dependent phosphoglycerate mutase was confirmed.

## Results

### The histidine phosphatase motif in Rv2135c

**Figure 1:**
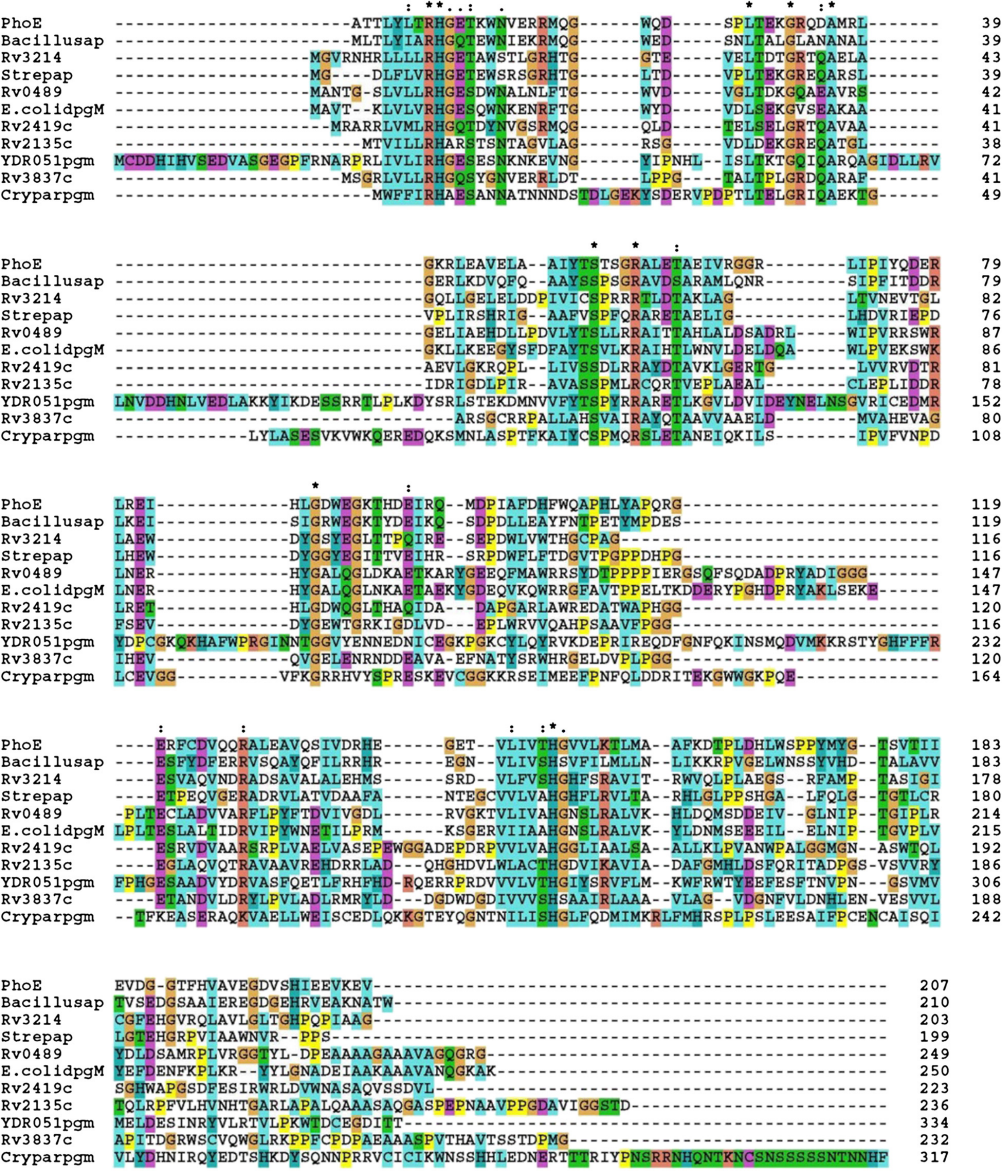
**Multiple alignment of amino acid sequences of some members of histidine phosphatase superfamily with Rv2135c.** The alignment was done with ClustalX2 using the default parameters. The asterisks indicate fully conserved amino acid residues of the superfamily. Colons indicate strongly similar residues, dots indicate weakly similar residues [37]. Bacillusap: Acid phosphatase of *Bacillus licheniformis.* Cryparpgm: Phosphoglycerate domain of *Cryptosporidium parvum.* E.colidpgM: Cofactor dependent phosphoglycerate mutase of *E. coli.* PhoE: Acid phosphatase of *Bacillus stearothermophillus.* Rv0489: Cofactor dependent phosphoglycerate mutase of *M. tuberculosis.* Rv2419c: Glucosyl-3-phosphoglycerate phosphatase of *M. tuberculosis.* Rv3214: Acid phosphatase of *M. tuberculosis.* Rv3837c: Probable cofactor dependent phosphoglycerate mutase of *M. tuberculosis.* YDR051pgm: Cofactor dependent phosphoglycerate mutase of *Saccharomyces arboricola.* Functions of Bacillusap, Cryparpgm and Rv3837c were predicted with bioinformatics while E.colidpgM, Rv0489, PhoE, Rv2419c, Rv3214 and YDR051pgm have been experimentally characterized.

Using NCBI BLAST [35], a number of proteins with similar sequences to Rv2135c were identified. Some sequences, including Rv0489, were aligned using ClustalX2 with the results shown in Figure 1. Most of the similar sequences contain the histidine phosphatase motif of ‘RHG’ , which contributes to catalysis, at the N-terminal region. The motif becomes ‘RHA’ (at residue 7–9) in Rv2135c. This is similar to the motif found in phosphoglycerate mutase domain containing protein of *C. parvum* (GAN CAD98474). Other conserved residues known to be involved in the catalysis of this superfamily from the analysis of others members are also present in Rv2135c. [4,9,36]. These include Arg57, Glu82, and a fully conserved His153 at the C-terminal region, Figure 1.

### Cloning and expression of C-His-Rv2135c and C-His-Rv0489

**Figure 2:**
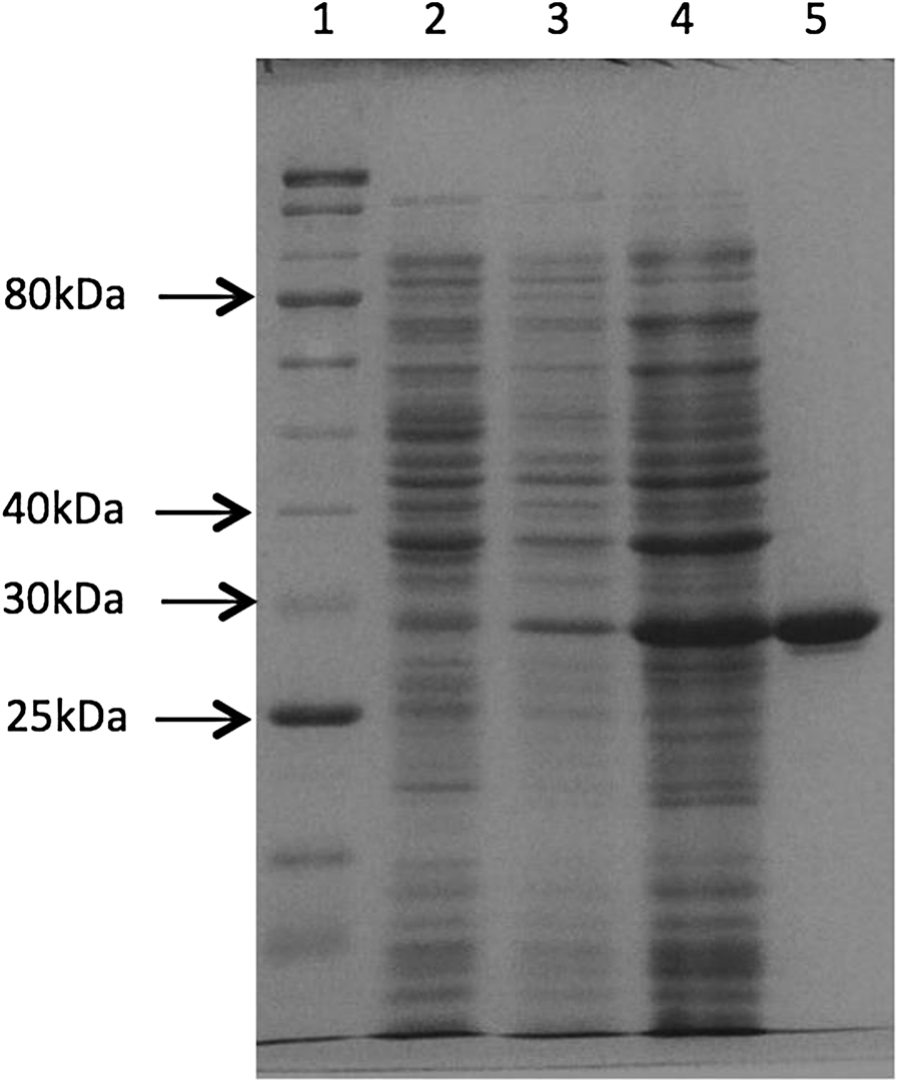
**12.5% SDS-PAGE of C-His-Rv2135c expressed in *E. coli *BL21(DE3) with or without induction and its purified form. Lane 1**: 5 μl of 10–250 kDa protein ladder (New England Biolabs). **Lane 2**: 10 μg of crude lysate of *E. coli* BL21(DE3) without any plasmid. **Lane 3**: 8.5 μg of crude lysate of *E. coli* BL21(DE3)-35c before induction with IPTG. **Lane 4**: 30 μg of crude lysate of BL21(DE3)-35c after induction with 0.4 mM IPTG for 8 hours at 25°C. **Lane 5**: 4 μg of recombinant C-His-Rv2135c eluted from IMAC column.

**Figure 3:**
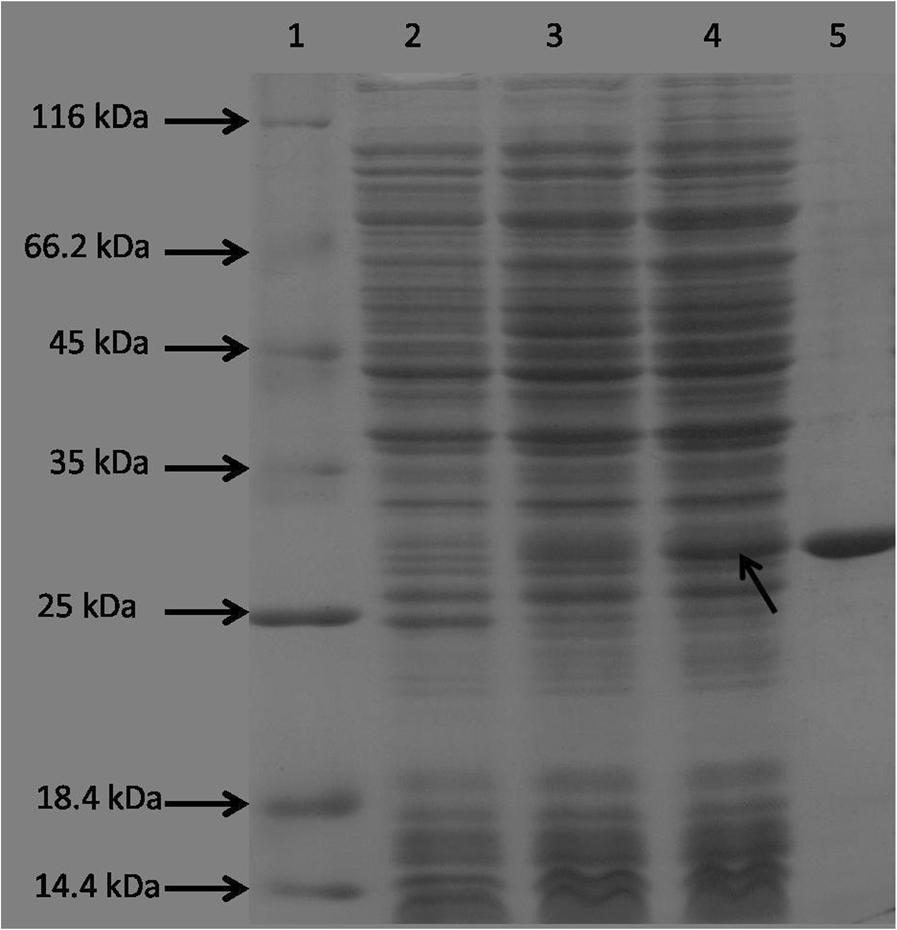
**12.5% SDS-PAGE of C-His-Rv0489 expressed in *E. coli *BL21(DE3) with or without induction and its purified form. Lane 1**: 9 μl of protein ladder (Fermentas SM0431). **Lane 2**: 15 μg of crude lysate of *E. coli* BL21(DE3) without any plasmid. **Lane 3**: 20 μg of crude lysate of *E. coli* BL21(DE3)-89 before induction with IPTG. **Lane 4**: 20 μg of crude lysate of BL21(DE3)-89 after induction with 0.03 mM IPTG overnight at 18°C. The arrow indicates the expressed recombinant protein, C-His-Rv0489. **Lane 5**: 3.5 μg of recombinant C-His-Rv0489 eluted from IMAC column.

*Rv2135c* and *Rv0489* genes of *M. tuberculosis* were successfully cloned with 6 histidine codons tagged at the 3′ end. The recombinant proteins were successfully expressed in *E. coli* BL21(DE3), resulting in appearance of extra protein bands with the sizes of about 27 kDa and 28 kDa in the soluble fraction of the cell lysates on SDS-PAGE. The sizes are in agreement with the amino acid calculated sizes of 25.95 kDa and 28 kDa respectively. C-His-Rv2315c and C-His-Rv0489 were purified to near homogeneity as shown in Figures 2 and 3, in a single step by loading into the cobalt charged resin column and eluting either by an increasing gradient of imidazole or fixed concentration of imidazole. The method resulted in about 40% yield and 2.4 folds increase in specific activity compared to the crude extract for C-His-Rv0489 as shown in Table 1. About 60% yield and 5.6 folds increase in specific activity compared to the crude extract for C-His-Rv2135c, when assayed at pH 5.8, were obtained as shown in Table 2.

**Table 1:** **The purification table of C-His-Rv0489c from 1 liter culture of *E. coli *** **BL21(DE3)-89c**

**Step**	**Protein (mg)**	**Activity (U)**	**Specific activity (U/mg)**	**Purification (fold)**	**Yield (%)**
Clarified extract	166.50	3696	22.20	1.00	100
Eluted fractions from IMAC	26.50	1432	54.00	2.40	38.70

The activities are reported using 3-phosphoglyceric acid as substrate.

**Table 2:** **The purification table of C-His-Rv2135c from 1 liter culture of *E. coli *** **BL21(DE3)-35c**

**Step**	**Protein (mg)**	**Activity (U)**	**Specific activity (U/mg)**	**Purification (fold)**	**Yield (%)**
Clarified extract	464	18.60	0.04	1.00	100
Eluted fractions from IMAC	50.40	11.60	0.23	5.60	62.40

The activities are reported using pNPP as substrate at pH 5.8.

### Enzymatic activities of C-His-Rv2135c and C-His-Rv0489

**Figure 4:**
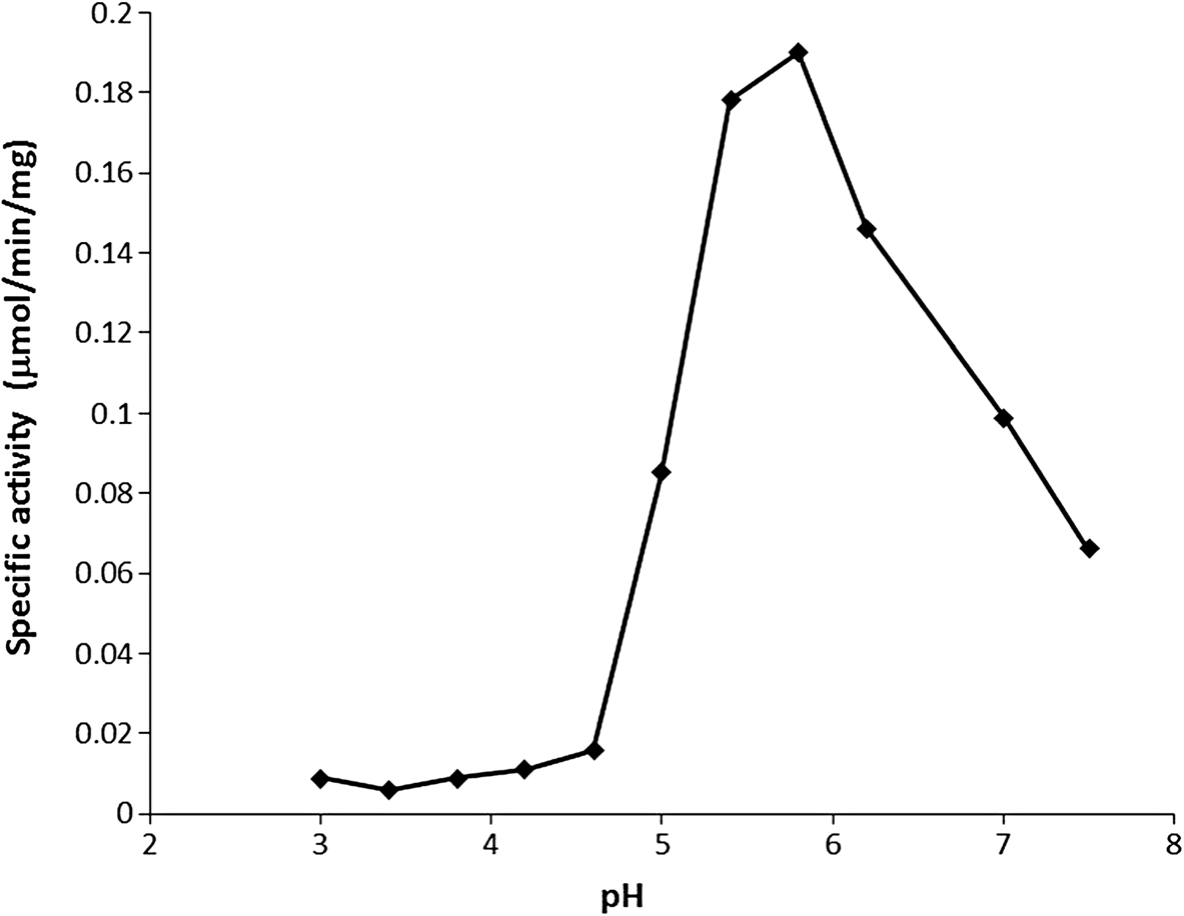
**The specific phosphatase activity of C-His-Rv2135c at different pH.** The optimal pH is 5.8.

C-His-Rv0489 showed clear phosphoglycerate mutase activity with specific activity of 54 μmol/min/mg. The kinetics of Rv0489 follows the Michaelis-Menten’s (see Additional file 1). The kinetic parameters of C-His-Rv0489 are shown in Table 3. In contrast, C-His-Rv2135c was found to possess no phosphoglycerate mutase activity but possesses acid phosphatase activity. The enzyme was assayed at pH 3.0, 3.4, 3.8, 4.2, 4.6, 5.0, 5.4, 5.8, 6.2, 7.0 and 7.5. The phosphatase activity was very low at pH 3.0-4.6, but was clearly observed at pH 5.0. It increased at pH 5.4 and peaked at pH 5.8. At higher pH, the activity decreased gradually as shown in Figure 4. Subsequent assays of C-His-Rv2135c were therefore done at the optimal pH of 5.8. A plot of the reaction velocities as a function of pNPP concentrations obeyed the Michaelis-Menten kinetics (see Additional file 1). The specific activity was estimated to be 0.23 μmol/min/mg.

**Table 3:** Kinetic parameters for the phosphoglycerate mutase activity of C-His-Rv0489

	**Km (mM)**	**kcat (min**^**-1**^**)**	**kcat/Km (mM**^**-1**^ **min**^**-1**^**)**
C-His-Rv0489	0.40 ± 0.02	250460 ± 8100	626100 ± 20300

**Figure 5:**
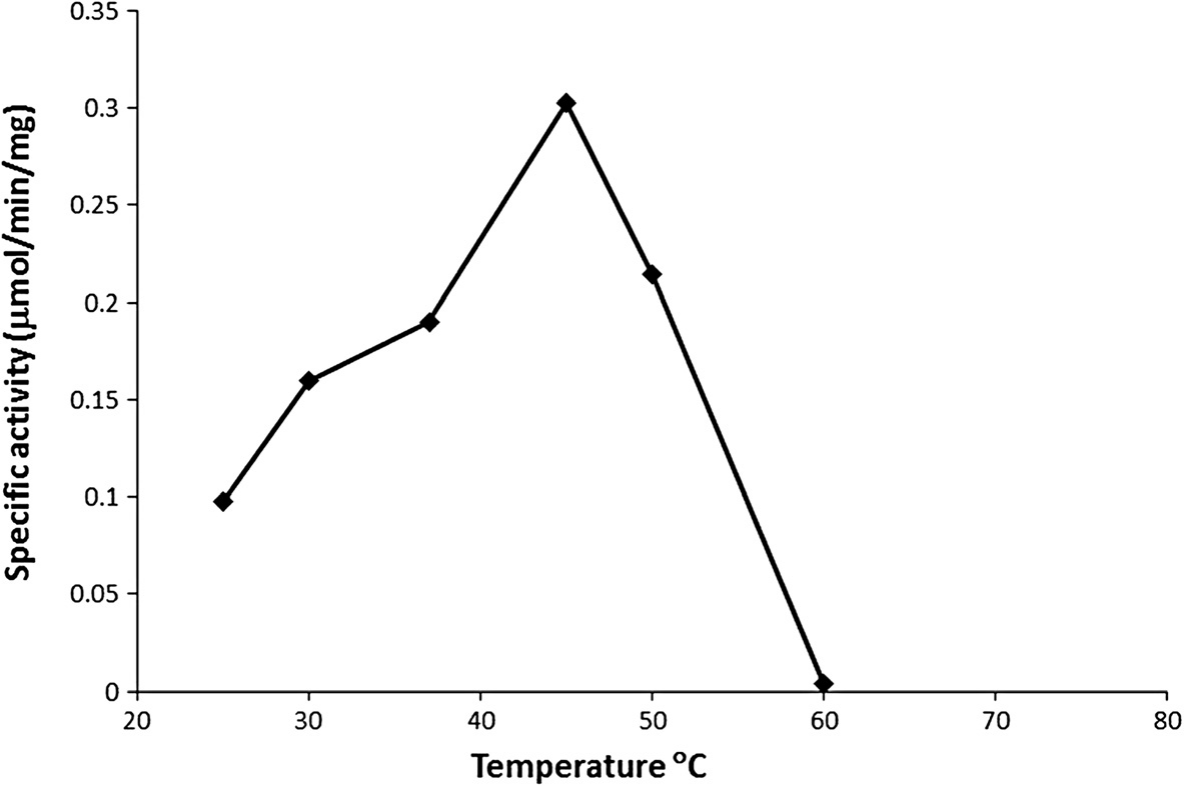
**The specific phosphatase activity of C-His-Rv2135c at different temperature.** The optimal temperature is 45°C.

The acid phosphatase activity of C-His-Rv2135c at pH 5.8 was determined at different temperatures. The maximum activity was found at 45°C as shown in Figure 5. This suggests that the structure of the enzyme is still relatively intact at 45°C. However, its activity dropped at higher temperatures, with no activity at all at 60°C. The kinetic parameters of C-His-Rv2135c are shown in Table 4.

**Table 4:** Kinetic parameters for the acid phosphatase activity of C-His-Rv2135c at pH 5.8 using pNPP as substrate

	**Km (mM)**	**kcat (min**^**-1**^**)**	**kcat/Km (mM**^**-1**^ **min**^**-1**^**)**
Rv2135c	10.60 ± 0.07	4170 ± 100	392 ± 10

### Substrates for C-His-Rv2135c

Using Malachite green assay, the amounts of phosphate groups hydrolyzed from different substrates in 25 mM citrate buffer at pH 5.8 were estimated, as shown in Table 5. No activity was detected for 3-phosphoglyceric acid, the substrate of phosphoglycerate acid mutase. In addition, the enzyme shows no activity with adenosine diphosphate (ADP) and glucose-6-phosphate. Its activities for fructose-6-phosphate, glycerol 1-phosphate and phosphoenolpyruvate were about the same and much less than the one for pNPP.

**Table 5:** Kinetic parameters for the activities of C-His-Rv2135c with different substrates at pH 5.8

	**Specific activity (mol/min/mg)**	**Km (mM)**
*p*-Nitrophenol Phosphate	0.23 ± 0.07	10.60 ± 0.07
Phosphoenolpyruvate	0.09 ± 0.002	11.25 ± 0.75
Glycerol-1-phosphate	0.05 ± 0.002	14.00 ± 0.00
ADP	0.00	
3-Phosphoglyceric acid	0.00	
Glucose-6-phosphate	0.00	
Fructose-6-phosphate	0.08 ± 0.009	7.75 ± 0.75

### Native molecular mass and stability

The size of the native form of C-His-Rv2135c was estimated by gel filtration to be 104.70 kDa. With the amino acid calculated size of 25.95 kDa, this suggests that C-His-Rv2135c forms a tetramer in the native state. This conforms to the results obtained by ND-PAGE, which provided the estimated native size of 103.85 kDa. The molecular mass of the native form of C-His-Rv0489 estimated from the gel filtration is 56.02 kDa. This indicates that C-His-Rv0489 forms a dimer, given both calculated and SDS-PAGE estimated molecular mass of the monomer of 28 kDa.

The acid phosphatase activity of C-His-Rv2135c at pH 5.8 was found to be enhanced by 15% in the presence of 10 mM magnesium ion. The enzyme was found to be stable in 50% glycerol at −20°C for up to 4 months with no significant change in activity.

## Discussion

In addition to Rv2419c [17] and Rv3214 [3] characterized recently, we have presented the study of a new mycobacterial phosphatase belonging to the histidine phosphatase superfamily. We report the first cloning, expression and characterization of Rv2135c, annotated as hypothetical in the genome database of *M. tuberculosis*[18]. Simple NCBI BLAST [35,38] reveals that most of the proteins similar to Rv2135c are annotated as hypothetical proteins or phosphoglycerate mutases. We demonstrated that C-His-Rv2135c possesses neither phosphoglycerate mutase nor phosphoglycerate phosphatase activity. However, it has phosphatase activity in acidic condition. Our findings support the necessity to experimentally characterize enzymes before their biochemical functions can be ascertained. This is important especially for the histidine phosphatase superfamily whose members can perform different metabolic functions [3,4,9,19].

C-His-Rv2135c has 6 more histidine residues at the C- terminal region than the native protein. The method of C-terminal tagging is commonly used for facilitating purification of enzymes and generally does not affect enzyme specificities. The specific acid phosphatase activity of C-His-Rv2135c (0.23 μmol/min/mg) is about 10 times less than that of Rv3214 (2.6 μmol/min/mg). However, some acid phosphatases of other pathogenic microorganisms are known to possess less specific activities than that of C-His-Rv2135c. Examples include the phosphatases of *Francisella tularensis* with specific activity of 0.002 μmol/min/mg [39] and *Entamoeba histolytica* with specific activity of 0.012 μmol/min/mg [40]. It should also be noted that the histidine phosphatase superfamily typically contains the characteristic motif ‘RHG’ at the N-terminal region. However, the motif present in Rv2135c is ‘RHA’ as found in the yet uncharacterized phosphoglycerate domain containing protein of *C. parvum* (GAN CAD98474). The replacement of glycine with alanine, another non-polar amino acid with a small side chain, may occur without any effect on the specificity of the enzymes in this family. Moreover, Rv2135c contains other residues reported to be important in the phosphatase activities of other members of the superfamily. These include Arg57, Glu82, and a fully conserved His153 at the C-terminal region [3,9,36]. Thus, we believe that Rv2135c performs an acid phosphatase function in its native environment.

The substrate specific to Rv2135c is unknown. Its sequence appeared to have little similarity to other previously annotated histidine phosphatases of *M. tuberculosis*[17], although the annotations of most of these phosphatases are still computational. Therefore there is no information suggesting the primary substrate of the enzyme. There are few experimentally characterized phosphatases in *M. tuberculosis*. These include Rv3214 and Rv2419c, which are histidine phosphatases [3,17], PtpA and PtpB which are tyrosine protein phosphatases [41,42], and PstP, a serine/threonine protein phosphatases [43]. The specific substrates of these phosphatases have not been identified yet, with the exception of Rv2419c, a glucosyl-3-phosphoglycerate phosphatase [17].

There are several known functions of histidine acid phosphatases, including extracellular metabolism, scavenging and regulatory functions. Rv2135c was identified as being associated with membrane protein fractions [20,44]. *M. tuberculosis* encounters a phosphate deficient acidic environment in an infected macrophage, and has been shown to depend on the acquisition of phosphate groups from the host environment for survival [29]. It is therefore intriguing to further study whether Rv2135c plays some roles in the intramacrophage environment, where it has been shown to be expressed [45].

Rv2135c and Rv2136c have been predicted to be in the same operon (http://genome.tbdb.org/annotation/genome/tbdb/). Rv2136c is the only mycobacterial gene with the catalytic motif of undecaprenyl pyrophosphate phosphatase. In bacteria, the enzyme hydrolyzes undecaprenyl pyrophosphate to produce undecaprenyl phosphate needed to translocate various cell wall intermediates from the cytosol across the cytoplasmic membrane for polymerization [46,47]. Despite the apparent essentiality of this function, undecaprenyl pyrophosphatases of many bacteria are known to be non-essential for their growth [48,49]. Rv2136c has also been shown to be non-essential for the survival of *M. tuberculosis*[50]. In some bacteria such as *E. coli*, other membrane associated phosphatases were shown to successfully perform the function of undecaprenyl pyrophosphatase when it was knocked out [49,51-53]. These include PgpB, YbjG and YeiU of *E. coli*, which belong to type 2 phosphatidic acid phosphatase family [53]. As Rv2135c and Rv2136c are predicted to be in the same operon, it may be possible that membrane associated Rv2135c performs a role similar to Rv2136c.

According to String Prokaryotic Operon Predictor (http://operons.ibt.unam.mx/OperonPredictor/), homologs of Rv2135c are identified in the same operon as the homologs of Rv2136c (undecaprenyl pyrophosphate phosphatase gene) in some other mycobacteria. These include *M. marinum, M. ulcerans, M. smegmatis* and *M. leprae*, but not *M. avium.* Using tblastx [35,38], it was found that homologs of Rv2135c and Rv2136c share adjacent positions in the genome of a number of other bacteria belonging to the actinomycetales such as *Nocardioides, Micrococcus, Cellulomonas, Geodermatophilus,* etc. Additional experiments are needed to investigate the functional relationship between these two genes.

Using Phyre2 [54], Rv2135c was modeled as a globular protein with a fairly large and hydrophobic pocket on its surface, which might provide a binding space for an undecaprenyl (see Additional file 2). A novel type of phosphoserine phosphatase of *Hydrogenobacter thermophiles*[55] was also identified as the most similar protein with known crystallographic structural data. However, the possible tetrameric structure of Rv2135c in the native form warrants further biochemical, computational and crystallographic studies in order to ascertain the natural substrate of this enzyme.

The crystal structure of Rv0489 was previously determined at 1.7 Å resolution. The residues at its active site were demonstrated to superimpose with corresponding residues of *E. coli* cofactor dependent phosphoglycerate mutase [16]. This study presents the first report of its biochemical activity and kinetic parameters, confirming it as a mycobacterial cofactor dependent phosphoglycerate mutase. Rv0489 was earlier found to be essential for the *in vitro* growth of H37Rv strain of *M. tuberculosis* by Himar1-based transposon mutagenesis [56], making it a putative target for drug development. Information about its kinetic parameters may be useful for formulating target-based screening assay for new drug discovery. This study shows that Rv0489 forms a dimer in solution. However, previous crystallization study carried out on Rv0489 showed it as a tetramer and referred to it as a dimer of dimers [16]. Cofactor dependent phosphoglycerate mutases from *E. coli* and *Homo sapiens* have been shown to be dimers [57,58] while those from *Saccharomyces cerevisae* and *Lactococcus lactis* are tetramers [59,60].

## Conclusion

Most well-characterized histidine acid phosphatases were reported from eukaryotes [9]. A bacterial histidine phosphatase is usually labeled as a phosphoglycerate mutase by automatic annotation systems. This is partly due to the much more abundance of bacterial phosphoglycerate mutases than bacterial histidine acid phosphatases in the databases. Orthologous proteins to Rv2135c, identified by reciprocal BLAST, are found widely in other mycobacteria as well as various taxa of bacteria, including *Staphylococcus aureus* and *E. coli*. Most of them are annotated as phosphoglycerate mutases or hypothetical proteins. It is possible that they are actually phosphatases. Experimental characterization of a sufficiently large number of bacterial histidine phosphatases will increase the accuracy of the automatic annotation systems towards a better understanding of this important group of enzymes.

## Methods

### Bacteria strains and culture conditions

*E. coli* strain DH5α was used for the maintenance and cloning of plasmids. Plasmid pET23b (Novagen, USA) was used as expression vector. It contains an inbuilt optional C-terminal hexahistidine tag for ease of protein purification. *E. coli* BL21 (DE3) was used as recipient hosts for recombinant protein expression [61]. *E. coli* was grown in Luria-Bertani (LB) medium. *M. tuberculosis* H37Ra (ATCC 25177) was grown on Middlebrook 7H11 agar supplemented with 10% Middlebrook OADC [Oleic acid Albumin Dextrose Catalase] Enrichment (Difco BBL, USA). *M. tuberculosis* genomic DNA was prepared as previously described [62].

### Identification of histidine phosphatase motif in Rv2135c

Using NCBI BLAST [35,38], Rv2135c protein was found to be similar to proteins of histidine phosphatase superfamily. Some of the similar proteins were aligned with Rv2135c using ClustalX2 with the default parameters [37]. The similar proteins included in the alignment are some experimentally characterized and predicted members of the superfamily. These are *M. tuberculosis* probable co-factor dependent phosphoglycerate mutase Rv0489 (GenBank accession number (GAN) CAE55288.1) [16], *E. coli* cofactor dependent phosphoglycerate mutase (E.colidpgM, Swissprot P62707), *PhoE* a broad specificity phosphatase from *B. stearothermophilus* (Protein data bank (PDB)1H2E_A) [63]*,* Rv3214, (GAN CAE55568) a *M. tuberculosis* acid phosphatase [3], an acid phosphatase from *Bacillus licheniformis* (Bacillusap, GAN EID46354), newly characterized glucosyl-3-phosphoglycerate phosphatase of *M. tuberculosis*, Rv2419c [17] (Swissprot P71724), and Rv3837c (GAN CAB06204) an uncharacterized paralog of Rv2135c. Members of histidine phosphatase superfamily from eukaryotes, the cofactor dependent phosphoglycerate mutase of *Saccharomyces arboricola* (YDR051pgm) (GAN EJS44264) and phosphoglycerate mutase domain containing protein of *Cryptosporidium parvum* (Cryparpgm) (GAN CAD98474) were also included.

### Cloning of Rv2135c and Rv0489

The open reading frame of Rv2135c and Rv0489 in the virulent strain H37Rv of *M. tuberculosis* is completely identical to the non-virulent strain H37Ra. The genomic DNA of H37Ra was, therefore, used as the template for the amplification of Rv2135c and Rv0489 by polymerase chain reaction (PCR) using primers 2135EF (AGCCGC*CATATG*ACCGTCATCTTGCTACG) and 2135CER(A*CTCGAG*GTCGGTGGAACCGCCGATC), and primers Rv0489-F (CTTGCC*CATATG*GCAAACACTGGCAGCCTGG) and Rv0489-R (CTCAAA*CTCGAG*CCCGCGGCCCTGGCCGGCCA) respectively. Recognition sequences of the restriction enzymes *Nde*I (CATATG) and *Xho*I (CTCGAG) are in italics. The PCR products were digested with *Nde*I and *Xho*I, ligated to appropriately digested expression plasmid pET23b with C-terminal histidine tag, and transformed into *E. coli* DH5α. Transformants were selected with ampicillin. Plasmids were purified from the transformants and sequenced to confirm the presence of correct genes tagged at the 3′ end with 6 histidine codons, designated as pET23b-35c and pET23b-89 respectively. *E. coli* BL21(DE3) strain was transformed with pET23b-35c and pET23b-89 to obtain strains BL21(DE3)-35c and BL21(DE3)-89 used for protein expression and from which the recombinant C-terminal histidine tagged proteins C-His-Rv2135c and C-His-Rv0489 were purified respectively.

### Expression and purification

A liter of LB medium with 100 μg/ml ampicillin was inoculated with an overnight culture of BL21(DE3)-35c to a final OD_600nm_ of about 0.03. The culture was incubated at 37°C with shaking speed of 200 rpm until OD_600nm_ reached about 0.6. The expression of the protein was then induced by the addition of IPTG to a final concentration of 0.4 mM. The culture was further incubated at 25°C at the shaking speed of 200 rpm for 8 hours. Cells were harvested by centrifugation at 3500 rpm at 4°C, washed with PBS pH 7.4 and stored at −20°C.

Similar treatment of BL21(DE3)-89 was done and resulted in precipitation of expressed protein after lysis. In order to obtain C-His-Rv0489 in the soluble fraction of cell lysate, BL21(DE3)-89 was cultured in the same media as above with the addition of 10% sucrose to OD_600nm_ of about 0.03. After the OD_600nm_ reached about 0.6, the expression of C-His-Rv0489 was induced with 0.03 mM of IPTG at 18°C overnight. Cells were harvested by centrifugation at 3500 rpm at 4°C, washed with PBS pH 7.4 and stored at −20°C.

Frozen cells were thawed on ice and suspended in the lysis buffer (20 mM Tris–HCl pH 7.0, 100 mM NaCl, 1 mM PMSF, 5 mM imidazole). The suspended cells were lysed by sonication using Misonix Sonicator 3000 (Qsonica LLC, USA) with 30 sec pause intervals until a clear lysate was obtained. The lysate was centrifuged at 11,000 rpm at 4°C for 20 min. The supernates, which contained the expressed histidine tagged protein, C-His-Rv2135c and C-His-Rv0489, were separated from other soluble proteins by immobilized metal affinity chromatography (IMAC). Briefly, the crude extracts were applied to cobalt charged resin column (Talon® Superflow column, GE Healthcare, Sweden) pre-equilibrated with the wash buffer (20 mM Tris–HCl pH 7.0, 100 mM NaCl, 5 mM imidazole). The column was then washed with 4 volumes of the wash buffer. For C-His-Rv2135c, the progress of purification was monitored by fast protein liquid chromatography (FPLC) using AKTA system (GE Healthcare, Sweden). Elution of the bound protein was done using 8 volumes of elution buffer I (20 mM Tris–HCl pH 7.0, 100 mM NaCl, containing a gradient of 0–60 mM imidazole). Eluted fractions were collected and loaded on SDS-PAGE to determine the purity of eluted proteins.

For C-His-Rv0489, after washing with 4 column volumes of lysis buffer, elution was done with elution buffer II (20 mM Tris–HCl pH 7.0, 100 mM NaCl, 150 mM of imidazole). The fractions with highest amount of recombinant C-His-Rv0489, determined by SDS PAGE were pooled and diluted to the imidazole concentration of 15 mM. The pooled fractions were then applied a second time to the cobalt charged resin column pre-equilibrated with wash buffer. The process of purification was repeated as the first column application to obtain pure C-His-Rv0489.

Purified C-His-Rv2135c and C-His-Rv0489 were concentrated using Amicon–Ultra 4 centrifugal filter unit (Merck Millipore USA) and stored in 20 mM Tris–HCl pH 7.0 containing 50% glycerol.

### Enzyme assays

Phosphoglycerate mutase activity: Phosphoglycerate mutase activities of C-His-Rv2135c and C-HisRv0489 in the 3-PGA to 2-PGA (forward) direction were monitored using an assay coupled to the oxidation of NADH as earlier described [64]. The assay was done in 500 μl of reaction mixture, containing 30 mM Tris–HCl pH 7.0, 20 mM KCl, 5 mM MgSO_4_, 1 mM ADP, 0.15 mM NADH, 0.2 mM 2,3-bisphophoglyceric acid, 2.5 U enolase (Sigma), 2.5 U pyruvate kinase (Sigma), 2.5 U lactate dehydrogenase (Sigma) [64] with ten concentrations of 3-phosphoglyceric acid (Sigma) (0.019, 0.039, 0.078, 0.156, 0.312, 0.625, 1.25, 2.5, 5 and 10 mM). Changes in absorbance at 340 nm using spectrophotometer (Thermo Electron Corporation, USA) were used in monitoring the oxidation of NADH. The values of absorbance of test solutions were corrected by the absorbance of the solution without enzymes. The assays were carried out in triplicate.

Acid phosphatase assay: The phosphatase activity was measured by monitoring the release of *p-*nitrophenol from *p-*nitrophenyl phosphate (pNPP) at a range of pH (3.0-7.5) as earlier described [64]. 25 mM sodium citrate buffer was used at pH 3.0-6.2 while 25 mM Tris–HCl was used at pH 7.0 and 7.5. The reaction, carried out at 37°C was started by the addition of the enzymes to the pre-warmed reaction buffer with eight concentrations of pNPP (New England Biolabs, USA) (0.78, 1.56, 3.125, 6.25, 12.5, 25, 50 and 100 Mm) in a total volume of 200 μl. The mixture was incubated for 60 min, and stopped with the addition of 600 μl of 1 N NaOH. Potato acid phosphatase (Sigma) was used as a positive control at pH 4.8 with 25 mM sodium citrate buffer. The amounts of released *p-*nitrophenol were estimated from the change in absorbance at 405 nm, corrected by the absorbance of the solution without the enzymes incubated at 37°C for the same period of time. All assays were carried out in triplicate.

Malachite green assay: The activities of C-His-Rv2135c with other substrates were investigated. The amount of phosphate group released from each of the substrates was detected by using the malachite green phosphate detection system (R&D Systems). It is based on quantification of the green complex formed between malachite green, molybdate and free orthophosphate as earlier described [65]. Phosphatase reaction was carried out in 25 mM sodium citrate buffer pH 5.8 at 37°C for 60 min in the presence of eight concentrations (0.78, 1.56, 3.125, 6.25, 12.5, 25, 50 and 100 mM) of glycerol-1-phosphate, glucose-6-phosphate, fructose-6-phosphate, adenosine diphosphate (ADP), phosphoenolpyruvate and 3-phosphoglyceric acid. The detection system was used according to the manufacturer’s instruction to detect the amount of released orthophosphate. The rapid color formation from the reaction was measured by the change in absorbance at 600 nm using a microplate reader (Glomax Multi Detection System, Promega, USA). The amounts of orthophosphate hydrolyzed were estimated in relation to a standard curve constructed with phosphate standard, according to the manufacturer’s instruction. All absorbance results were corrected for enzyme-unrelated absorbance change and all assays were carried out in triplicate.

Estimation of the kinetic parameters: The rate constants (Km) were estimated using Michaelis-Menten kinetics by plotting the values of reaction rates obtained against the concentrations of substrates. The curves were fit non-linearly by generalized reduced gradient (GRG) solving method using the Solver add-in in Microsoft Excel. Km was determined for each experiment and averaged. The specific activities, turnover numbers (kcat) and the catalytic efficiencies (kcat/Km) were estimated using Michaelis-Menten kinetics.

### Determination of molecular mass

The native molecular mass of C-His-Rv2135c was determined under non-denaturing condition by gel filtration chromatography and native polyacrylamide gel electrophoresis (ND-PAGE) while gel filtration only was used for the determination of the molecular mass of C-His-Rv0489 in solution.

Pre-packed 10 mm X 30 cm column of Superdex 200 HR 10/30 equilibrated in 20 mM sodium phosphate buffer, pH 7.0, containing 0.1 M NaCl was used with four standard protein markers: catalase (232 kDa), lactate dehydrogenase (140 kDa), bovine serum albumin (66 kDa) from Sigma and MPT83 (50 kDa) [66], a mycobacterial protein purified in our laboratory. Proteins were eluted at the buffer flow rate of 0.2 ml/min. The void volume of the column was determined by loading blue dextran unto the column. A standard curve was constructed by plotting the molecular masses versus the ratio Ve/Vo for the standard protein markers, while Ve is the volume of elution of each protein and Vo is the void volume of the column. The Ve/Vo for C-His-Rv2135c and C-His-Rv0489 were used in determining their molecular weight from the standard curve.

ND-PAGE was done as previously described [67]. Briefly, 4 μg of the purified protein and standard proteins were loaded on four native gels of different acrylamide concentrations. The concentrations of the acrylamide in resolving gels were 6, 7, 8 and 9%. 3% acrylamide was used for stacking in each resolving gel. The relative migrations of the purified protein and the standard proteins in each gel, designated as R_f_, were estimated from each gel [67] by dividing the migration distance of the protein standards by the migration distance of the dye front. 100log(R_f_X100) values for each protein standard and C-His-Rv2135c were plotted against the gel concentrations. The negative slope obtained for the standard protein was plotted against their molecular weight values to obtain a standard curve. The molecular weight of C-His-Rv2135c was estimated from the standard curve.

## Competing interests

We the authors hereby declare that there is no conflict of interest concerning this manuscript.

## Authors’ contributions

OOC, PP and SW conceived the study. OOC cloned Rv2135c and carried out the purification and biochemical characterization of the two enzymes. PS cloned Rv0489 and participated in the purification of the enzymes. KR and OOC determined the molecular masses of the purified enzymes. TP and SW supported the research. OOC and PP wrote the manuscript. PP coordinated and critically revised the manuscript. All authors read and approved the manuscript.

## Supplementary Material

Additional file 1 **Reaction rates of C-His-Rv2135c and C-His-Rv0489.** This file contains a Microsoft Word document showing the actual reaction rates for the phosphatase activity of C-HisRv2135c (Table 1S) and the phosphoglycerate mutase activity of C-His-Rv0489 (Table 2S) for three different experiments .The quality of the curves from which the rate constants (km) and the maximum velocities (Vmax) were estimated are shown in Figure 1S and Figure 2S. Click here for file

Additional file 2 **Phyre2 modeling of Rv2135c.** This file contains the pdb document detailing the modeling of Rv2135c monomer with Phyre 2 program. The file can be opened with iSilo program. Click here for file
