# Supplementary material for: Functional characterization of two members of histidine phosphatase superfamily in Mycobacterium tuberculosis
Source: BMC Microbiol. 2013 Dec 11;13:292. doi: 10.1186/1471-2180-13-292 (PMC3866925; doi:10.1186/1471-2180-13-292)
Supplement: Additional file 1 — Reaction rates of C-His-Rv2135c and C-His-Rv0489. This file contains a Microsoft Word document showing the actual reaction rates for the phosphatase activity of C-HisRv2135c (Table 1S) and the phosphoglycerate mutase activity of C-His-Rv0489 (Table 2S) for three different experiments .The quality of the curves from which the rate constants (km) and the maximum velocities (Vmax) were estimated are shown in Figure 1S and Figure 2S. [file 1471-2180-13-292-S1.docx]

Table 1S. The phosphatase activity of C-His-Rv2135c at pH 5.8 with different concentrations of pNPP substrate. Column 2-4 show the reaction rates from 3 different experiments.

| **pNPP**  **(mM)** | **Reaction rate**  **(μmol/min/mg)**  **(1)** | **Reaction rate**  **(μmol/min/mg)**  **(2)** | **Reaction rate**  **(μmol/min/mg)**  **(3)** |
| --- | --- | --- | --- |
| 100 | 0.199 | 0.202 | 0.202 |
| 50 | 0.199 | 0.206 | 0.234 |
| 25 | 0.152 | 0.161 | 0.194 |
| 12.5 | 0.124 | 0.107 | 0.130 |
| 6.25 | 0.090 | 0.090 | 0.090 |
| 3.125 | 0.051 | 0.049 | 0.061 |
| 1.56 | 0.024 | 0.022 | 0.032 |
| 0.78 | 0.007 | 0.012 | 0.011 |

**Figure 1S.** A fitted curve showing the relationship between the rate of reaction C-His-Rv2135c in column 2 of table 1S and the concentrations of pNPP.

Curving fitting to the Michaelis-Menten equation by the least square error was done by generalized reduced gradient (GRG) nonlinear solving method using Solver add-in of MS Excel. V = Vmax / (1 + (Km/[S]), where V is the reaction rate, Vmax is the maximum reaction rate, [S] is the concentration of substrate and Km is the Michaelis-Menten constant. The reported Km is the average from three experiments.

Table 2S. The mutase activity of of C-His-Rv0489 with different concentrations of 3-phosphoglyceric acid (3-PGA) substrate. Column 2-4 show the reaction rates from 3 different experiments.

| \| **3-PGA Concentration**  **(mM)** \|  \| **Reaction rate**  **(μmol/min/mg)**  **(1)** \| **Reaction rate**  **(μmol/min/mg)**  **(2)** \| **Reaction rate (µmol/min/mg)**  **(3)** \| \| \| --- \| --- \| --- \| --- \| --- \| --- \| \| 2.5 \|  \| 47.17 \| 42.88 \| 45.02 \| \| 1.25 \|  \| 46.63 \| 43.42 \| 45.02 \| \| 0.625 \|  \| 45.56 \| 35.91 \| 40.74 \| \| 0.312 \|  \| 28.94 \| 20.90 \| 24.66 \| \| 0.156 \|  \| 18.22 \| 11.79 \| 15.01 \| \| 0.078 \|  \| 8.58 \| 5.36 \| 6.97 \| |  |
| --- | --- | --- | --- | --- | --- | --- | --- | --- | --- | --- | --- | --- | --- | --- | --- | --- | --- | --- | --- | --- | --- | --- | --- | --- | --- | --- | --- | --- | --- | --- | --- | --- | --- | --- | --- | --- | --- |

**Figure 2S.** A fitted curve showing the relationship between the rate of reaction C-His-Rv0489 in column 4 of table 2S and the concentrations of 3-PGA.

Curving fitting to the Michaelis-Menten equation by the least square error was done by generalized reduced gradient (GRG) nonlinear solving method using Solver add-in of MS Excel. V = Vmax / (1 + (Km/[S]), where V is the reaction rate, Vmax is the maximum reaction rate, [S] is the concentration of substrate and Km is the Michaelis-Menten constant.

The reported Km is the average from three experiments.

.
